# Exploring the Characteristics of Soil Nonvolatile Metabolites From Geographical Locations in Guizhou Using UPLC Fingerprint Profiling

**DOI:** 10.1155/jamc/5197541

**Published:** 2025-10-09

**Authors:** Jie Zhang, Jinxiu Luo, Wei Jiang, Ruijuan Zhao, Yechun Lin, Dongqing Xu, Wenjing Zhu, Weichang Gao, Kai Cai

**Affiliations:** ^1^Upland Flue-Cured Tobacco Quality & Ecology Key Laboratory of CNTC, Guizhou Academy of Tobacco Science, Guiyang 550081, China; ^2^College of Agronomy and Biotechnology, Southwest University, Chongqing 400715, China; ^3^Zunyi Branch of Guizhou Tobacco Company, Zunyi 563100, China; ^4^Guizhou Provincial Tobacco Quality Supervision & Test Station, Guiyang 550025, China

**Keywords:** chemometrics, geographical locations, soil nonvolatile metabolite, tobacco flavor styles, UPLC fingerprint profiling

## Abstract

Soil metabolites are not only the integrated expression of soil biological activities but also the pivotal drivers of biogeochemical cycling, thereby significantly influencing the formation of crop quality. This study established an improved ultra-performance liquid chromatography (UPLC) fingerprint with chemometrics for characterizing the nonvolatile metabolite profiles of soils from geographical locations defined by different tobacco flavor styles in Guizhou. The homogenization extraction and then UPLC analysis were selected as the optimal system due to their superior repeatability and reproducibility, with intraday and interday RSD% of the common peaks (retention time and peak area) below 2.75%. The fingerprint profiling was established using 18 soil samples from three locations, namely the honey-sweet region I, II, and the fresh-sweet region. Thirty common peaks were identified, with similarities ranging from 0.809 to 0.956. Then, the common peaks were subjected to chemometrics analysis. These results indicated that significant differences were observed by principal component analysis (PCA), and 17 characteristic metabolites were viewed as primarily discriminatory by partial least squares discriminant analysis (PLS-DA). The total content of characteristic metabolites followed a trend of honey-sweet region II > honey-sweet region I > fresh-sweet region, with individual metabolites generally higher in the honey-sweet region. Finally, external validation using the hierarchical cluster analysis (HCA) and Fisher discriminant analysis (FDA) model accurately classified four soil samples, further confirming the representativeness of the characteristic metabolites. This study supplies a theoretical foundation to understand the relationship between tobacco flavor formation and soil metabolism, showing great potential applications in agricultural research.

## 1. Introduction

The flavor styles of tobacco raw materials were a critical basis for stabilizing product quality and forming characteristic flavor profiles [1]. The tobacco aromatic attributes exhibited substantial variability, primarily influenced by the synergistic effects of ecological conditions and genetic factors. Guizhou Province is located in the low-latitude mountain plateau of southwestern China with a rising altitude from east to west, which leads to distinct ecological zones. These geographical variations induce substantial differences in tobacco leaf quality, chemical composition, and aromatic constituents across different altitudinal belts, ultimately leading to diverse tobacco flavor styles [2]. As a fundamental ecological determinant, soil conditions significantly influence tobacco quality characteristics and flavor formation mechanisms [3].

Soil organic matter (SOM) is the basic substrate for the provision of ecosystem services that fuel the metabolism behind soil function [4]. Because of spatial and temporal variations, there were regional differences in SOM composition [5]. The previous study demonstrated the total content of active SOM from three regions defined by tobacco flavor styles following a trend of fresh-sweet flavor > partial fresh-sweet flavor > middle flavor. Additionally, medium- and high-active SOM also showed significant differences among the three regions [6]. By influencing the transcription and biosynthesis processes of sesquiterpenoids and chlorophyll, active SOM significantly increased the concentration of aromatic constituents in tobacco leaf. It suggested that the content and distribution profiles of active SOM may be important factors for the formation of tobacco flavor styles [7].

Soil nonvolatile metabolites (small organic molecules), as critical components of active SOM, cover a diverse array of chemical classes, such as amino acids, peptides, lipids, and carbohydrates [8, 9]. These metabolites are a resultant (indirect) output of the biological hierarchy (genome, transcriptome, and proteome), and they represent the manifestation of material cycling within biological communities [10]. Consequently, the composition and distribution characteristics of nonvolatile metabolites in soil can decode the roles of small-molecule organic compounds in biochemical systems related to regulating and supporting ecosystem services. This serves as a critical pathway to elucidate the intricate linkages between soil metabolism and their ecological environments [11].

As an integrative analytical platform, a fingerprint is a method on the basis of overall chemical signatures obtained by multiple analytical tools, which can effectively explain sample-specific chemotaxonomic signatures within biological matrices [12]. When synergized with chemometrics of multidimensional feature extraction, this methodology demonstrates remarkable efficacy for certification, identification of origin, and quality evaluation of traditional herbs, foods, and agricultural products, among others. As evidenced by GC-IMS-based volatile compound fingerprint profiling of American ginseng with different origins, there were marked differences between the volatile components, and then partial least squares discriminant analysis (PLS-DA) successfully identified origin-specific markers [13]. Furanic derivatives fingerprint profiling of sugarcane honey with UHPLC-PDA analysis, coupled with chemical pattern recognition, achieved regional traceability [14]. For quality evaluation of the Changshan Huyou flower, flavonoid fingerprint profiling with HPLC analysis was used to discriminate samples with different harvest times [15]. In the field of the tobacco industry, fingerprint profiling has also been utilized for tracing the tobacco's geographical origins with pyrolysis products, monitoring flavor quality with volatile components, and distinguishing tissue-specific mapping with chlorogenic acid derivatives [16, 17]. However, the literature lacks the utilization of fingerprints for nonvolatile metabolites in tobacco-planting soil, which could elucidate the effects of soil ecological mechanisms on the formation of tobacco flavor styles and conduct soil traceability.

According to the four dimensions of ecological environment, sensory evaluation, tobacco chemistry, and metabolism [18], tobacco-planting regions were divided into two major flavor styles in Guizhou, namely, honey-sweet in the central and eastern regions and fresh-sweet in the western region. In this study, a straightforward, validated, and repeatable methodology was developed to establish the characteristic ultra-performance liquid chromatography (UPLC) fingerprint of soil nonvolatile metabolites in different geographical locations defined by tobacco flavor styles in Guizhou. First, the sample extraction and chromatographic conditions were optimized to obtain more target peaks and achieve high sensitivity. Then, the fingerprints were constructed with common peaks, and the chemometrics were successfully applied for the identification of geographical locations. Finally, the correlation between characteristic metabolites and physicochemical properties was investigated, and geographical location traceability functions were verified with external validation. This study provided a novel theoretical foundation for screening characteristic tobacco regions and exploring the relationship between tobacco flavor formation and soil metabolism.

## 2. Materials and Methods

### 2.1. Soil Sampling and Preparation

The honey-sweet styles are widely distributed in central and eastern regions of Guizhou, with diversity in ecological conditions. Previous studies have shown that tobacco in the eastern regions showed stronger caramel-sweet styles, which was a significant difference from honey-sweet [19]. Hence, this study collected 18 samples from different geographical locations in Guizhou tobacco-planting soil and classified them into three regions, namely honey-sweet region I (MT I, numbered S1∼S6, from central regions, Zunyi), honey-sweet region II (MT II, numbered S7∼S12, from eastern regions, Tongren), and fresh-sweet region (QT, numbered S13∼S18, from western regions, Xingyi). Additional samples (numbered S19∼S22) with external validation were randomly collected from geographical locations in Wengan (central region), Huangping (central region), Bozhou (central region), and Weining (western region). The types were yellow soils.

To make the soils more comparable, the bulk soil in tobacco-planting sampling was initiated on October 1, 2023, and ended on October 30, 2023, which is the postharvest stage of tobacco. Plough-layer soil was sampled from points by a stainless-steel auger, excluding the surface litter. All samples were collected using the five-point sampling method, and then soils were placed in a zip-locked plastic bag, immediately stored in ice packs, and transported to the laboratory [20]. A part of the soil samples was freeze-dried in a vacuum freeze dryer (SIM Inc., DE, USA) and then stored at −70°C for nonvolatile metabolite analysis. The other part was dried at room temperature for analyzing the soil physicochemical properties. The dried soils were crushed and ground into a fine powder via a mortar and pestle, and then the soil powders were passed through a 180-μm stainless-steel sieve for further analysis. Determination of soil physicochemical properties, namely total phosphorus (TP), total nitrogen (TN), total potassium (TK), available nitrogen (AHN), available phosphorus (AP), available potassium (AK), organic matter (OM), and pH, was followed by that proposed by Zhu et al. [21, 22].

### 2.2. Metabolite Extraction

To prevent the metabolites from being adversely affected by decomposition due to excessive temperatures, soil samples were collected, extracted, and analyzed at low temperatures. A 0.5000 g aliquot of soil and 1.0 mL of precooled (4°C) HPLC purity acetonitrile (ACN) were added in a 2.0 mL microcentrifuge tube. The extraction of nonvolatile metabolites was homogenized into a 4°C freezing grinder (Jing Xin Technology, China) at a shaking frequency of 60 Hz for 5 min. This extraction procedure was repeated three times with a total of 15 min. Then, the microcentrifuge tubes were centrifuged at 12,000 rpm for 5 min at 4°C. The supernatant was carefully transferred to a 2 mL microcentrifuge tube and then dried under gentle N_2_ flow. The residuals were redissolved with 100 μL of 50% methanol–water (*v/v*) solution. All the extracts were kept in 2-mL vials and preserved in a refrigerator before UPLC analysis.

### 2.3. UPLC Analysis

UPLC profiling was executed on a Waters UPLC Acquity system (Milford, MA, USA) coupled with a PDA eλ detector. The whole configuration was controlled by Waters Empower 3 Software. The separation column was BEH UPLC C18 (2.1 × 100 mm, 1.7 μm) maintained at a temperature of 30°C. The mobile phase was composed of water (solvent A) and ACN (solvent B) according to the following gradient: from 0 to 11 min, a gradient from 2% to 90% B; from 11 to 14 min, a gradient from 90% to 100% B; and finally from 14.1 to 17 min, a gradient from 100% to 2% B. The temperature for the sample manager is set to 4°C, and the injection volume is 5 μL. The PDA detection wavelengths were set at 230 nm. Compared with UPLC chromatograms between blank and QC samples, the retention time of nonvolatile metabolites was mainly distributed from 4 to 12 min; all peaks within this range were integrated as target peaks for UPLC fingerprint profiling analysis.

### 2.4. Statistical Analysis

The data, presented as means ± standard deviation, were analyzed using SPSS 22.0 (IBM®, IL, USA). To assess significant differences in soil physicochemical properties within each region, one-way ANOVA with the Duncan test at *p* < 0.05 was employed. The principal component analysis (PCA) was carried out using the web platform MetaboAnalyst 5.0 (https://www.metaboanalyst.ca/). The hierarchical cluster analysis (HCA) and PLS-DA were using the software SIMCA 14.1. The performance of PLS-DA models was assessed by goodness-of-fit (*R*^2^*Y*) and the goodness-of-prediction (*Q*^2^) parameters. The higher the *R*^2^*Y* and *Q*^2^ parameters, the higher the model performance, accepting a *Q*^2^ cutoff > 0.5 as good predictability. The significance of the characteristic metabolites was ranked using the variable importance in projection (VIP) score (1.0) from the PLS-DA [23]. Correlation analyses were performed using the *corrplot* package in R statistical software (R Studio) to explore the relationships between nonvolatile metabolites and physicochemical properties. Figures and tables were generated with Origin 8.5 (OriginLab®, MA, USA) and Microsoft Excel.

## 3. Results

### 3.1. Change of Soil Physicochemical Properties

As shown in Table 1, the content of TN ranged from 1.55 to 1.95 g kg^−1^, with a significant difference between MT II and QT (*p* < 0.05). TP ranged from 0.83 to 1.12 g kg^−1^, TK ranged from 18.63 to 19.45 g kg^−1^, and AP ranged from 30.68 to 37.10 mg kg^−1^, with no significant difference across the three regions (*p* > 0.05). The content of AHN ranged from 109.92 to 141.02 mg kg^−1^, with a significant difference between MT II and QT (*p* < 0.05). AK ranged from 307.00 to 709.33 mg kg^−1^, with a significant difference between MT I and QT (*p* < 0.05). The soil pH ranged from 6.11 to 7.56, with significant differences across the three regions (*p* < 0.05), while OM ranged from 31.47 to 43.22 g kg^−1^, with no significant difference (*p* > 0.05).

**Figure 1:**
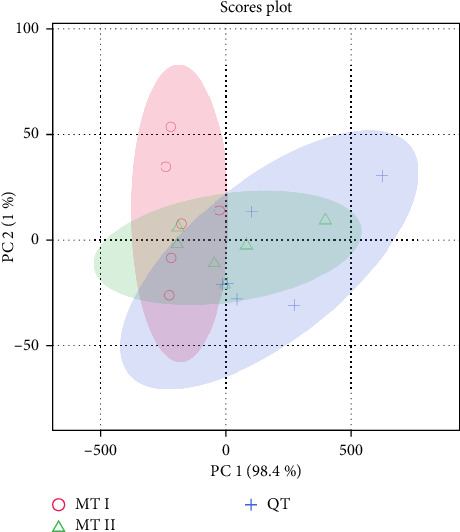
The PCA score plot is presented for different geographical locations. In the figure, the geographical locations are represented with colors: red for MT I, green for MT II, and blue for QT.

To systematically compare the soil physicochemical properties from different geographical locations, PCA was conducted to discriminate differences.  Figure 1 shows that the first two principal components could cumulatively explain 99.4% of total variance through the first two PCs (PC1: 98.4%; PC2: 1.0%), which could represent the overall physicochemical properties characteristics. However, each group could not be distinguished among them, especially the MT II and QT, which suggested physicochemical properties exhibit substantial homogeneity across geographically discrete sites. These results indicated that the conventional soil physicochemical properties were unable to characterize the changes from geographical locations.

### 3.2. Optimization of Chromatographic and Pretreatment Conditions

The separation efficiency of soil metabolites was compared with isocratic elution in 90% ACN and water and gradient elution in ACN ranging from 10% to 90% and water [24]. The results indicated that gradient elution achieved better separation for target compounds with different polarities. The column temperature ranging from 25°C to 45°C had an insignificant impact on the separation efficiency; thus, the commonly used temperature of 30°C was selected as the separation condition. When the injection volume was 5 μL, the solvent effect on the chromatographic peaks was minimal, and the peak intensity of the target peaks was higher. Using PDA full-wavelength scanning between 210 and 500 nm, the optimal wavelength of 230 nm was determined, which minimized the impact of baseline noise and resulted in higher sensitivity.

The selection of extraction solvents represents a critical factor for obtaining high extraction efficiency. The commonly used three organic solvents ACN, methanol (MeOH), and ethanol (EtOH) were employed for soil nonvolatile metabolite extraction. Analytical results demonstrated similar extraction efficiencies for EtOH and MeOH, while ACN exhibited superior performance through enhanced chromatographic peak resolution and peak area. Further, the aqueous ACN solutions (80%, 60%, and 50%) revealed peak intensity decline with increasing solvent polarity, confirming the polarity of nonvolatile organic metabolites is relatively weak. Based on these findings, ACN was selected as the optimal solvent.

**Figure 2:**
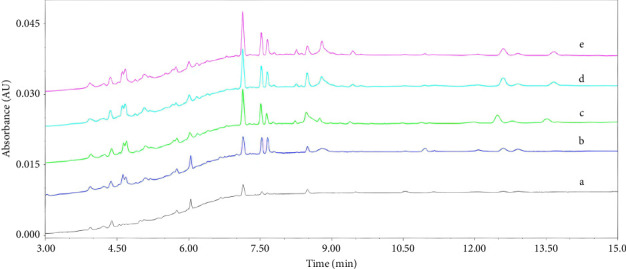
UPLC chromatogram of QC samples extracted under different homogeneous conditions. In the figure, the treatment conditions are represented with letters: (a) for 30 Hz, 3 min cycle^−1^; (b) for 60 Hz, 1 min cycle^−1^; (c) for 60 Hz, 3 min cycle^−1^; (d) for 60 Hz, 5 min cycle^−1^; (e) for 60 Hz, 10 min cycle^−1^.

The optimization of extraction procedures involved a comparative evaluation of three conventional techniques: ultrasonication (35 min at 50 Hz, ≤ 30°C), homogenization (three 3 min cycles at 60 Hz, 4°C), and vortex mixing (5 min at 2500 rpm). The ultrasonication required prolonged extraction time, and vortex mixing exhibited limitations in solvent-sample interaction and poor reproducibility. Consequently, homogenization was selected as the optimal extraction procedure. The homogenization extraction of frequency and time was further optimized to maximize the extraction efficiency. Figures 2(a), 2(b), and 2(c) reveal the peak intensity was dependent on the enhancement of frequency, achieving superior performance at 60 Hz versus 30 Hz. Figures 2(b), 2(c), 2(d), and 2(e) indicate the extraction efficiency was increased with extraction time and then kept constant at 5 min cycle^−1^. Therefore, the optimal parameters were three repeated extractions of 5 min cycles at 60 Hz. To further improve the method sensitivity, the extraction solution was concentrated to dry under nitrogen and then redissolved with methanol–water (*v/v*), which showed a significant increase in peak intensity without altering the composition of the nonvolatile metabolites.

### 3.3. Establishment of UPLC Fingerprint Profiling

UPLC fingerprint profiling was established to evaluate the soil nonvolatile metabolites from geographical locations. First, the UPLC fingerprint method was verified by reproducibility and repeatability of retention time and peak intensity. Repeatabilities (intraday relative standard deviation, or RSD%) were calculated by analyzing five independent QC samples within a single day, while reproducibilities (interday RSD%) were calculated over three consecutive days. The intraday precision of the target peaks' retention time (*T*) and areas (*S*) was T_RSD%_ < 1.71% and S_RSD%_ < 2.21%; the interday precision was T_RSD%_ < 2.14% and S_RSD%_ < 2.75%, indicating that the reproducibility and repeatability were excellent; therefore, it could be used to construct a fingerprint profiling of soil nonvolatile metabolites.

**Figure 3:**
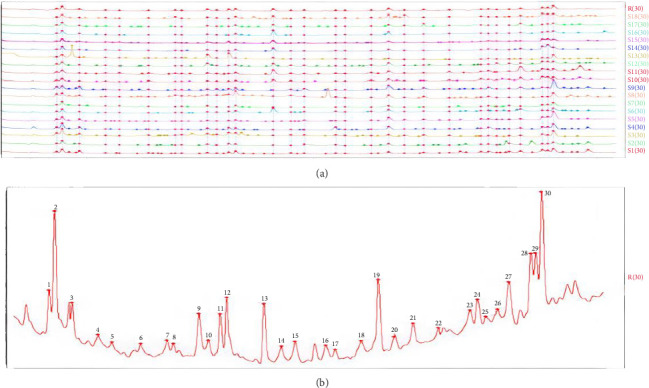
Overlay of UPLC chromatograms of 18 samples from geographical locations (a). Reference fingerprint in UPLC chromatogram of nonvolatile metabolites (b).

To construct the fingerprint profiling, it is very important to include more abundant compounds presented in every sample [25]. Before analyzing fingerprints, chromatograms of different samples must be normalized. The normalization process involved in selecting common peaks in the chromatograms and normalizing the retention times of all common peaks; 30 common peaks were corrected by multipoint correction and peak matching (Figure 3(a)), and obtained a UPLC reference fingerprint (Figure 3(b)).

The similarity assessment can be reached by computation of the related coefficient from the original data [26]. Similarity analysis was conducted between 18 soil samples and the reference chromatograms. The similarity index was calculated using the average fusion vector method. The related coefficient between each chromatogram and the simulative average chromatogram is shown in Table 2. The correlation coefficient is more closed to 1, the more similarity is achieved. The similarity indexes ranged from 0.809 to 0.956, which indicated a higher degree of consistency in the chemical composition across soil samples with different geographical locations, but there were differences in their content distribution.

### 3.4. Chemical Pattern Recognition for Characteristic Metabolites

**Figure 4:**
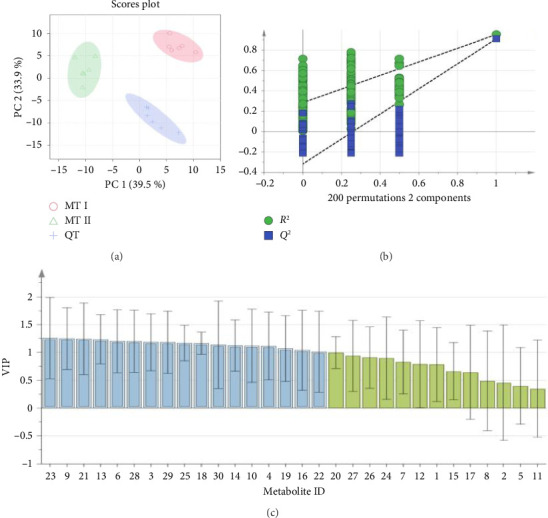
PCA score plot (a); PLS-DA score plot (b); and VIP plot of 30 common peaks (c).

Based on the multivariate UPLC fingerprint profiling, PCA was performed to visualize the variance between soil samples. The resulting score plot is shown in Figure 4(a); the first component described 39.5% of the variance, and the second component 33.9%, yielding a total variance of 73.4%, which could represent the overall characteristics of the fingerprint profiling. The 18 soil samples were clustered into three categories, indicating that the distribution of soil metabolite contents from the three geographical locations of MT I, MT II, and QT varied significantly.

To expand the classification differences and screen characteristic metabolites associated with the difference between the three regions, the supervised PLS-DA was used. The models, with *R*^2^*Y* = 0.954 and *Q*^2^ = 0.919, indicated a stable and dependable model. A permutation test (200 iterations) was used for internal verification, confirming no overfitting of the model (Figure 4(b)). The results revealed an intercept of 0.283 for the *R*^2^ fitted straight line on the *Y*-axis, further affirming the model's reliability. Additionally, the intercept of −0.318 for the *Q*^2^ fitted straight line on the *Y*-axis suggested that the constructed model is not overfitted, ensuring its validity and applicability. The characteristic soil metabolites were screened as significant differences from geographical locations defined with three tobacco flavor styles, based on VIP values > 1.0, *p*-values < 0.05 (Figure 4(c)). Seventeen characteristic metabolites were found as peaks 3, 4, 6, 9, 10, 13, 14, 16, 18, 19, 21, 22, 23, 25, 28, 29, and 30, respectively.

**Figure 5:**
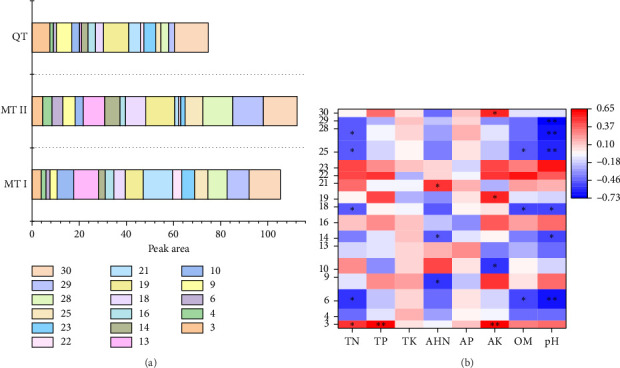
Characteristic metabolite peak areas distribution map (a), correlation heat map between characteristic metabolite and physicochemical properties (b).

The distribution of peak areas for characteristic metabolites from different regions is shown in Figure 5(a). The results indicated that the total content of characteristic metabolites in MT II was significantly higher than that in other regions, specifically exhibiting a trend of MT II > MT I > QT regions. The content of each characteristic metabolite in the soil from MT I, II was mostly higher than those from QT, with peaks 4, 6, 10, 13, 14, 18, 25, 28, and 29, while peaks 3 and 9 were significantly lower. This suggested that the honey-sweet region contain a richer abundance of soil nonvolatile metabolites. Significant differences were also observed between MT I and II. Specifically, the content of peaks 10, 13, 16, 21, 22, 23, and 30 was significantly higher in soil samples from the MT I, whereas the content of peaks 4, 6, 9, 14, 18, 19, 25, 28, and 29 was significantly lower. Only the content of peak 3 did not exhibit a significant difference. In addition, the correlation analysis between each characteristic metabolites and physicochemical properties (Figure 5(b)) showed that peak 3 was significantly positively correlated with TN, TP, and AK (*p* < 0.05), peaks 6, 18, and 25 were significantly negatively correlated with TN, OM, and pH (*p* < 0.05), peak 29 was significantly negatively correlated with TN and pH (*p* < 0.05), most of the peaks were significantly negatively correlated with pH.

### 3.5. Geographical Locations Traceability Functions and Verification

**Figure 6:**
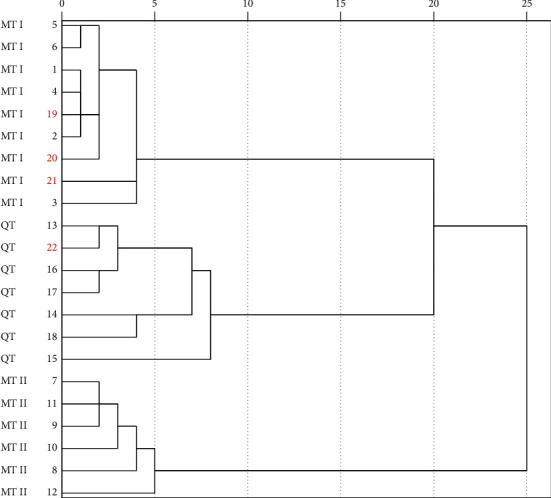
External validation pedigree diagram of soil samples. The red boxes are external samples.

Four external soil samples, designated as S19 to S22, were randomly selected for validating external sample traceability. The soil variation in changes from geographical locations was better illustrated by an HCA map with Euclidean distance [27]. In this analysis (Figure 6), the peak areas of 17 characteristic metabolites from the original 18 soil samples (S1 to S18) and the four external samples served as variables. The four external samples were plainly divided into different groups according to the dendrogram. Specifically, cluster I for MT I included samples S1∼S6 and S19∼S21, cluster II for MT II contained samples S7∼S12, and cluster III for QT involved S13∼S18 and S22. Consistent with the above PCA results, the clustering map could clearly distinguish changes in geographical locations, showing the robust representativeness of the 17 characteristic metabolites identified through fingerprint profiling.

Fisher discriminant analysis (FDA) seamlessly integrated information from different data sources, reducing the inherent limitations of individual data sources and improving the overall performance and generalization ability of the model [28]. To further validate the discriminatory capability of the 17 characteristic soil metabolites in tracing the geographical origins, the Fisher quantitative discriminant function model was employed, which comprised three discriminant functions: Y1 for MT I, Y2 for MT II, and Y3 for QT in equations (1), (2), and (3). Utilizing the peak areas of these 17 metabolites in the 18 soil samples as key indicators, we conducted both original discriminant analysis and cross-validation on the samples.  Table 3 shows the model accuracy constructed using the FDA algorithm. The model generated from the fused dataset performed well with 100% discrimination accuracy, and the four external soil sample data followed with excellent accuracy, with 100% accuracy on the training set and an average cross-validation accuracy of 95.5%.

(1) $$\begin{array}{c} Y_{1}=43.811X_{3}-11.177X_{4}-46.433X_{6}-20.953X_{9}+18.292X_{10}-17.462X_{13}-50.305X_{14}+6.327X_{16}+3.618X_{18}-40.839X_{19}+27.402X_{21}-11.695X_{22}-10.305X_{23}-59.721X_{25}-32.981X_{28}+32.284X_{29}-6.127X_{30}-88.429, \end{array}$$

(2) $$\begin{array}{c} Y_{2}=-344.321X_{3}-185.526X_{4}+92.928X_{6}+204.429X_{9}+149.402X_{10}+171.361X_{13}+292.261X_{14}-150.172X_{16}+10.565X_{18}+270.771X_{19}-132.852X_{21}+54.1X_{22}-52.712X_{23}+134.148X_{25}+478.65X_{28}-135.442X_{29}-138.132X_{30}-787.894, \end{array}$$

(3) $$\begin{array}{c} Y_{3}=238.804X_{3}+173.393X_{4}-19.953X_{6}-148.286X_{9}-151.577X_{10}-124.429X_{13}-185.832X_{14}+120.585X_{16}-13.708X_{18}-179.583X_{19}+78.642X_{21}-31.335X_{22}+58.43X_{23}-38.2X_{25}-367.867X_{28}+74.586X_{29}+110.522X_{30}-342.565. \end{array}$$

## 4. Discussion

### 4.1. Soil Metabolomics Analysis Using UPLC Fingerprint Profiling

SOM is composed of any substance initially produced by organisms in the soil and is a universal substrate on which most life on earth relies for survival. It was considered a complex mixture of low molecular weight organic compounds (LMWOC) [10]. A new perspective suggested that the majority of SOM, especially the dissolved fraction (DOM), was composed of small molecules produced by the breakdown of cells and released metabolites by plants and microorganisms [29]. The microbial exo-metabolome and intracellular metabolome, as a branch of dissolved organic matter (DOC) pools, were a rich source of information that provided functional insights into soil processes [30]. To delve into the role of OM in soil nutrient status and quality as well as plant-microbe metabolic interactions [31, 32], GC/LC-MS targeted and untargeted metabolomics has been used for the analysis of small molecule metabolite composition in soil matrices [33]. However, this technology has challenges such as instrument cost, matrix effect, and metabolite identification. In our study, a fingerprint profiling approach integrated with chemometrics was employed to distinguish differences by identifying common compounds in a complex background. The nonvolatile metabolite distribution was obtained using soil UPLC fingerprints from typical tobacco planting regions in Guizhou. Then the correlation analysis indicated that soil physicochemical properties had different effects on the characteristic metabolites. The soil physicochemical properties can directly modulate metabolites through adsorption and desorption and may also indirectly alter metabolite profiles by influencing microbial communities. The interactions between nitrogen-fixing bacteria and AHN are closely linked to a variety of soil metabolites, including flavonoids, organic acids, and carbohydrates [34]. SOM, TN, AHN, AK, and AP had positive correlations with *Nitrospira* and *Sphingomonas*, which also could obviously influence the soil metabolites [35]. Furthermore, the identified characteristic metabolites distinguished soil variations between diverse geographical locations. To sum up, the UPLC-PDA method was both economical and straightforward, laying a solid foundation for future research into the molecular mechanisms for the formation of tobacco leaves' flavor styles using metabolomics.

### 4.2. ACN as an Optimal Solvent for Nonvolatile Metabolite Extraction in Soil

To date, a series of available extraction solvents has been used for different SOM fractions. The two most common methods were to use water (i.e., biologically available) or organic solvents (check for intracellular, available, and soil adsorption/absorption), or a combination of both, for recovering metabolites from the soil. The selection of solvent will greatly affect the recovered compounds [36, 37]. In this study, it was found that similar extraction efficiencies for EtOH and MeOH, while ACN exhibited superior performance. ACN, with an index polarity of 5.8, can extract hydrophilic lignin and tannin compounds effectively, which contain high oxygen numbers. The composition of soil nonvolatile metabolites, including polyphenols, organic acids, polyphenolic tannin-like compounds, and others, with high oxygen-to-carbon (O/C) ratios (O/C > 0.6) and low hydrogen-to-carbon (H/C) ratios (H/C < 1) was reported for higher extraction efficiency in ACN [38]. Furthermore, ACN was also good at separating the target compounds as a mobile phase. The UPLC isocratic elution conditions in 90% ACN revealed that the majority of peaks emerged within 3.5 min and poor chromatographic resolution was obtained. Conversely, gradient elution conditions resulted in better separation with varying polarities.

### 4.3. Soil pH May Drive the Variation in Nonvolatile Metabolites

Unlike soil physicochemical properties, the nonvolatile metabolites exhibited significant variations among different tobacco planting regions, indicating that metabolites were highly sensitive to changes in the soil environment and were better suited for characterizing variations in soil ecological factors. The content of characteristic metabolites in most soil samples from MT was substantially higher than that of QT. The correlation analyses revealed a strongly negative correlation between pH and characteristic metabolites. The pH could affect the activity and diversity of soil microorganisms and the stable humus component, which could increase or decrease metabolite content [39]. The stable humus component increases evidently under slightly acidic soil conditions, which is beneficial to the accumulation of soil metabolites to affect the formation of aroma and taste flavor [40]. Furthermore, pH can also alter the adsorption-desorption capacity between metabolites and soil [41]. These mechanisms result in soil pH, which may drive the variation in nonvolatile metabolites. It can also be inferred that the slightly acidic soil conditions are conducive to the formation of honey-sweet tobacco leaves.

### 4.4. Unraveling the Relationship Between Nonvolatile Metabolites and Tobacco Flavor Styles

The formation of honey-sweet tobacco leaves necessitated richer soil nonvolatile metabolite conditions. Different from the typical honey-sweet flavor style, tobacco in the eastern regions (MT II) showed stronger caramel-sweet styles. The total content of characteristic metabolites in MT II was significantly higher than MT I. Some studies showed that soil metabolites (e.g., amino sugar, proteins, lipids, tannins, and carbohydrates) affected the chemical composition of tobacco leaves, thereby affecting the formation of tobacco flavor styles [42, 43]. Trehalose and *γ*-aminobutyric acid (GABA) as key soil metabolites can directly influence plant physiological growth through root uptake. GABA can increase the contents of phenylalanine (the synthetic substrate of flavonoids). The expression activity of phenylalanine ammonia-lyase (PAL), cinnamic acid 4-hydroxylase (C4H), and 4-coumarate coenzyme A ligase (4CL) was also improved. These phenomena improved phenylalanine metabolism to promote the entry of its substrate into the flavonoid pathway and then increase flavonoids [44]. Tobacco aroma components were derived from these secondary metabolites, which were closely related to the formation of tobacco aroma styles [45]. Moreover, soil microorganisms participate in a lot of key processes of soil ecosystems, including nutrient cycling, OM turnover, and soil structure maintenance, which are believed to significantly impact the production of secondary metabolites in tobacco leaf [46]. As a critical variable of SOM, soil metabolites could play a role in the transformation and cycling of organic matter and plant nutrients in soil and can function as a sink (during immobilization) or source (during mineralization) of labile nutrients [47]. It has been shown that the lack of OM in soil could impact microbial community structure and biomass and may also lead to a decrease in the activity of various enzymes, thereby affecting the production of secondary metabolism in plants [48, 49], thereby influencing the flavor profile of tobacco in various geographical locations. Further, the correlation among the tobacco flavor style, the content of different metabolites, and microbial life activities, as well as the metabolic interactions between plants and microorganisms, will be investigated.

## 5. Conclusion

A UPLC-PDA has been developed for fingerprint profiling to explore the characteristics of soil nonvolatile metabolites from geographical locations in Guizhou. The results revealed significant variations in soil nonvolatile metabolite content among the MT I, II, and QT regions. Seventeen characteristic metabolites were viewed as primarily distinguishing in three regions. The total content of the characteristic metabolites followed the trend MT II > MT I > QT, with individual metabolites consistently higher in the honey-sweet region compared to the fresh-sweet region. Additionally, HCA and FDA models were constructed based on these characteristic metabolites, and external validation from different regions was successfully identified with a recognition rate of 95.5%. These results suggest that UPLC-PDA has great potential in tracing the geographical origins of soil metabolism related to flavor formation. Overall, UPLC-PDA has great potential for the geographical origin traceability of soil metabolism associated with flavor formation, showing great potential applications in agricultural research. Further, high-resolution mass spectrometry should be used to identify the metabolites and analyze the function mechanism.

## Figures and Tables

**Table 1:** Changes in soil physicochemical properties from different geographical locations.

Region	TN (g kg^−1^)	TP (g kg^−1^)	TK (g kg^−1^)	AHN (mg kg^−1^)	AP (mg kg^−1^)	AK (mg kg^−1^)	OM (g kg^−1^)	pH
MT I	1.87^a^ ± 0.32^ab^	0.83 ± 0.26^a^	18.63 ± 4.75^a^	141.02 ± 24.48^ab^	37.10 ± 21.33^a^	307.00 ± 59.25^a^	39.38 ± 10.48^a^	6.69 ± 0.56^a^
MT II	1.55 ± 0.30^a^	0.90 ± 0.30^a^	19.12 ± 7.47^a^	109.92 ± 17.79^a^	33.77 ± 18.28^a^	462.00 ± 249.08^ab^	31.47 ± 7.51^a^	6.11 ± 0.76^b^
QT	1.95 ± 0.29^b^	1.12 ± 0.35^a^	19.45 ± 9.33^a^	120.92 ± 25.50^b^	30.68 ± 13.22^a^	709.33 ± 264.30^b^	43.22 ± 10.00^a^	7.56 ± 0.36^c^

*Note:* According to Duncan's multiple range test (*p* < 0.05), different letters in the same column indicate significant differences.

^a^The data shown are the mean ± SD.

**Table 2:** Similarities of chromatograms obtained from geographical locations of 18 soil samples.

No.	Related coefficient
S1	0.956
S2	0.934
S3	0.937
S4	0.942
S5	0.918
S6	0.918
S7	0.933
S8	0.841
S9	0.907
S10	0.916
S11	0.818
S12	0.936
S13	0.809
S14	0.845
S15	0.922
S16	0.893
S17	0.883
S18	0.922

**Table 3:** The accuracy of the model constructed using the Fisher discriminant analysis algorithm.

Event	Category			Total	Validation accuracy (%)
	MT I	MT II	QT		
Training set	6	0	0	6	100
	0	6	0	6	100
	0	0	6	6	100

Cross-validation	9	0	0	9	100
	0	6	0	6	100
	1	0	6	7	85.7

External validation	3	0	0	3	100
	0	0	1	1	100

## Data Availability

The data that support the findings of this study are available from the corresponding authors upon reasonable request.
